# Balancing Water Uptake, UV Visible Screening, and Mechanical Strength in Cellulose Alginate Quercetin Hydrogel Films

**DOI:** 10.1021/acsomega.6c01788

**Published:** 2026-05-25

**Authors:** Sarah Kalli Silva da Silva, Andre Lamounier Caixeta, Marlon Bender Bueno Rodrigues, Bruna Bicca Fernandes, Patricia Oliveira Schmitt, Lincoln Audrew Cordeiro, Ivandra Ignes de Santi, Evandro Piva, Neftali Lenin Villarreal Carreno, Amanda Dantas de Oliveira, Cesar Aguzzoli, Everton Granemann Souza, Andre Luiz Missio, Chiara das Dores do Nascimento

**Affiliations:** † Graduate Program in Materials Science and Engineering (PPGCEM), 37902 Federal University of Pelotas (UFPel), Pelotas, RS 96010-610, Brazil; ‡ Graduate Program in Dentistry (PPGO), School of Dentistry, Federal University of Pelotas (UFPel), Pelotas, RS 96015-560, Brazil; § Graduate Program in Materials Science and Engineering (PPGMAT), University of Caxias do Sul, Caxias 95070-560, Brazil; ∥ Graduate Program in Electronics and Computer Engineering (MEEC), 67714 Catholic University of Pelotas (UCPel), Pelotas, RS 96015-560, Brazil; ⊥ Graduate Program in Sciences and Technologies in Education (PPGCITED), Federal Institute of Education, Science, and Technology of Rio Grande do Sul (IFSul), Pelotas, RS 96060-290, Brazil

## Abstract

Combining high water affinity and strong UV–vis shielding while preserving mechanical performance in fully biobased cellulose films remains challenging, as hydrophilic polysaccharide modifiers and polyphenolic additives can disrupt network cohesion, promote phase-enriched domains, and trigger early fracture. Here, renewable hydrogel films were engineered from bleached *Eucalyptus* cellulose by incorporating sodium alginate (SA) and quercetin (Q) as multifunctional additives to tune structure–property relationships. Composite hydrogels were homogenized under a standardized and scalable protocol and converted into continuous films by doctor blade coating. Film morphology, intermolecular interactions, crystallinity, optical response, wetting and hydration, and tensile behavior were assessed by FE-SEM, ATR-FTIR, XRD, UV–vis spectroscopy, contact-angle kinetics, gravimetric water uptake, and tensile testing. Quercetin governed the visible color shift and enabled strong UV–vis attenuation (>95%), whereas alginate improved film continuity and enhanced hydration. FTIR and XRD supported predominantly noncovalent incorporation, consistent with a physically cross-linked polysaccharide network. An optimum formulation, CEL-2SA-1Q (97/2/1 wt %), delivered the highest mechanical performance (*F*_max_ = 147.85 ± 9.82 N, UTS = 67.20 ± 4.46 MPa, and Young’s modulus = 1411.3 ± 18.3 MPa), corresponding to ∼1.5× higher *F*_max_ and UTS and 2.75× higher modulus than neat cellulose films. The same formulation showed enhanced hydrophilicity, with the contact angle decreasing to ∼35° within 60 s and a 24 h water uptake about 3-fold higher than neat cellulose. These combined attributes suggest the potential of the optimum film as a prospective fully biobased, high-absorbency, UV–vis-opaque liner for sustainable packaging of high-moisture, light-sensitive products, where exudate retention and photoprotection are critical for preserving appearance and delaying oxidation.

## Graphical abstract

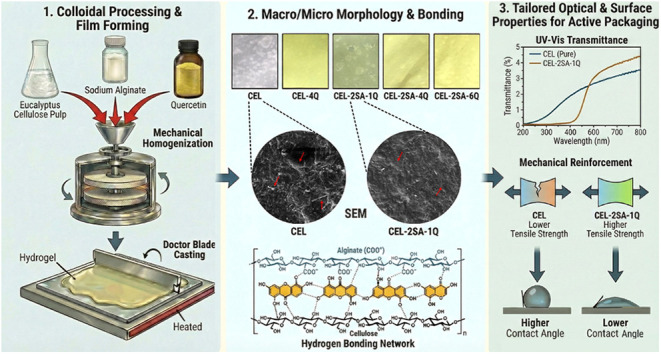

## 1 Introduction

The growing global demand for sustainable industrial processes, aligned with the United Nations Sustainable Development Goals (SDGs), particularly SDG 12, has accelerated the replacement of synthetic polymers with biodegradable and renewable materials.^1,2^ Conventional plastics such as PE, PP, PS, PET, and PVC persist in the environment and contribute to microplastic accumulation.^3–5^

Two main strategies have been explored: partial substitution using blends and composites, and full substitution using biobased matrices. The first approach combines biodegradable polymers with reinforcing phases such as cellulose, improving stiffness and strength while maintaining processability.^6–10^ However, it does not eliminate fossil-based components and may still contribute to environmental issues.^11^

The second strategy focuses on fully biobased systems, where cellulose stands out due to its abundance, renewability, and ability to form functional films. In these systems, performance is governed by network architecture and intermolecular interactions such as hydrogen bonding and electrostatics.^12,13^

A major limitation of cellulose-based films is the intrinsic trade-off between water uptake and mechanical strength. Enhancing hydration often leads to plasticization and loss of stiffness, making simultaneous optimization challenging.^13,14^

To address this, polyphenols have been incorporated into cellulosic matrices due to their ability to modulate hydrogen bonding and introduce additional functionality. However, these systems often suffer from phase heterogeneity and reduced mechanical performance at higher loadings.^15–17^

Another widely explored approach is combining cellulose with ionic polysaccharides such as alginate or chitosan, exploiting complementary interactions and hydration behavior.^18,19^ These systems rely on noncovalent interactions, which are attractive for green chemistry but can also lead to structural heterogeneity and mechanical limitations.

A promising alternative is a ternary cellulose-sodium alginate-quercetin system, in which each component performs a complementary function. Cellulose provides the structural framework, alginate increases water affinity, and quercetin enhances intermolecular interactions while imparting optical functionality.^18,20,21^

Related systems have been reported mainly as active films, but they often show limited mechanical strength. For example, cellulose-alginate films containing glycerol typically reach 18.03–22.4 MPa.^22^ In contrast, the optimized ternary formulation developed here showed markedly improved tensile strength, together with enhanced water uptake and UV–vis shielding. These findings suggest that an appropriate structural design may mitigate the usual trade-off between mechanical performance and functionality.

Accordingly, this work investigates cellulose-based composite films containing sodium alginate and quercetin, with emphasis on how these additives affect structure, intermolecular interactions, and final performance. The aim is to obtain a balanced combination of mechanical strength, hydration capacity, and optical shielding for sustainable packaging applications.

## 2 Materials and Methods

### 2.1 Materials

The cellulose used in this study was a bleached *Eucalyptus* spp. pulp supplied by CMPC (Chile), in the form of cellulose board sheets, with a moisture content of approximately 3.6%. According to technical data reported by the manufacturer for bleached eucalyptus kraft pulps, intrinsic viscosity values are typically above 700 mL/g, corresponding to a viscosity-average degree of polymerization (DPv) in the range of approximately 2500–3300. The cellulose density was considered to be in the range of 1.5–1.6 g/cm^3^, consistent with literature values for cellulose I. Analytical-grade sodium alginate (C_6_H_7_NaO_6_)_*n*_, with nominal purity of 90% (CAS No. 9005–38–3), was purchased from Dinâmica Química Contemporânea Ltd. (Brazil). Analytical-grade quercetin dihydrate (C_15_H_10_O_7_·2H_2_O), supplied as a powder (CAS No. 6151–25–3), was purchased from Inlab Confiança (Brazil).

### 2.2 Pretreatment for Hydrogel Preparation

To ensure adequate component dispersion, structural homogeneity, and batch-to-batch reproducibility, all formulations were processed using a standardized pretreatment protocol while keeping the total solids content constant across samples. Five cellulose-based composite hydrogels containing sodium alginate and quercetin were prepared according to the compositions listed in Table 1.

**Table 1:** Formulations of Cellulose-Based Composite Hydrogels^a^

Sample	Description	Cellulose (wt %)	Sodium alginate (wt %)	Quercetin (wt %)
CEL	Neat cellulose hydrogel (control)	100	0	0
CEL-4Q	Cellulose hydrogel with 4 wt % quercetin	96	0	4
CEL-2SA-1Q	Cellulose/alginate hydrogel with 1 wt % quercetin	97	2	1
CEL-2SA-4Q	Cellulose/alginate hydrogel with 4 wt % quercetin	94	2	4
CEL-2SA-6Q	Cellulose/alginate hydrogel with 6 wt % quercetin	92	2	6

a Compositions expressed as weight percent (w/w) relative to the total solids in each formulation. Total solids were fixed at 20 g per batch (1% m/v in 2 L of water).

First, the moisture content of each solid component (cellulose, sodium alginate, and quercetin) was determined, and the weighed masses (analytical balance, Shimadzu AUW220D, Kyoto, Japan; 0.0001 g readability) were corrected to a dry mass basis. The amounts of each component were then calculated so that each formulation contained 20 g of total solids, corresponding to a fixed final concentration of 1% (m/v) in 2 L of distilled water. This normalization enabled direct comparability among formulations and allowed the effects of composition to be assessed independently of overall solids loading.

### 2.3 Doctor Blade Coating Technique for Film Preparation

Hydrogel films were produced using a doctor blade coating system (T CAST, AutoCoat, São Paulo, Brazil), following the procedure reported by LaChance et al.^23^ Due to the low aqueous solubility and aggregation tendency of quercetin, the hydrogel formulations were homogenized using a high-shear rotor-stator homogenizer (Ultra-Turrax, IKA T25 digital, IKA-Werke GmbH & Co. KG, Staufen, Germany) at 12,000 rpm for 10 min prior to the doctor blade coating step. After homogenization, each hydrogel formulation was poured onto a glass plate maintained at 55 °C and placed on the instrument bed to promote initial adhesion and improve layer uniformity.

Coating was performed with the doctor blade gap set to 8 mm and the coating speed set to 10 mm/s. Three consecutive layers were deposited (approximately 50 mL per layer), with 30 min intervals between depositions to allow leveling. After the final deposition, the coated plates were kept at 65 °C until the films released from the glass substrate, yielding continuous films with uniform macroscopic appearance. The films were then stored in a desiccator until physicochemical and morphological characterization.

### 2.4 Characterization

#### 2.4.1 Field Emission Scanning Electron Microscopy (FE-SEM)

Film morphology was examined by field emission scanning electron microscopy (FE-SEM) using a MIRA4 LMS microscope (TESCAN, Brno, Czech Republic). Micrographs were acquired under high vacuum at an accelerating voltage of 30 kV. Prior to imaging, specimens were mounted on aluminum stubs and sputter-coated with a thin conductive layer to minimize charging.

#### 2.4.2 Wettability

Surface wettability was evaluated by static contact angle measurements using the sessile drop method. A Theta Lite TL100 optical tensiometer (Biolin Scientific, Gothenburg, Sweden), operated with OneAttension software, was used for all measurements. A 10 μL droplet of distilled water was gently placed on the film surface using a motorized syringe, and wetting was recorded for 60 s at 20 frames s^–1^. The apparent contact angle, θ­(*t*), was determined by fitting the droplet profile with the Young–Laplace model. For each formulation and surface, at least three droplets were analyzed on independent specimens prepared under identical conditions, and results are reported as mean ± standard deviation.

#### 2.4.3 Water Uptake

Water uptake was determined following ASTM D570 with adaptations for thin films. Square specimens (1 cm × 1 cm) were cut from each film and dried in a ventilated oven at 40 °C for 24 h. The initial dry mass (*M*_i_) was measured using an analytical balance (0.0001 g readability). Specimens were fully immersed in distilled water at room temperature and removed after 1, 2, 4, 17, and 24 h. At each time point, samples were retrieved using tweezers and handled carefully to avoid mechanical damage. Excess surface water was gently removed with absorbent paper, and the wet mass (*M*_f_) was recorded. Water uptake (WU%) was calculated using eq 1

1 $$\mathrm{WU}\;\left(\%\right)=\frac{M_{f}-M_{i}}{M_{i}}\times 100$$

For each formulation and immersion time, at least three specimens were tested, and results are reported as mean ± standard deviation.

#### 2.4.4 UV–Vis Spectroscopy

Optical properties were evaluated by UV–vis spectroscopy using a Shimadzu UV-2600 spectrophotometer (Shimadzu Corporation, Kyoto, Japan). Transmittance and absorbance spectra were recorded from 250 to 800 nm at room temperature, with the films positioned perpendicular to the incident beam.

#### 2.4.5 Fourier Transform Infrared Spectroscopy (FTIR)

Infrared spectra were collected by attenuated total reflectance Fourier transform infrared spectroscopy (ATR-FTIR) using a Shimadzu Prestige-21 spectrometer (Kyoto, Japan). Measurements were acquired as the average of 90 scans over the range 600–4000 cm^–1^ with a spectral resolution of 4 cm^–1^, following ASTM E1252–13.

#### 2.4.6 X-ray Diffraction (XRD)

X-ray diffraction (XRD) patterns were collected using a Rigaku MiniFlex-600 diffractometer (Rigaku Corporation, Akishima, Tokyo, Japan) with Cu Kα radiation (λ = 0.15406 nm), operating at 40 kV and 15 mA. Data were acquired over a 2θ range of 10–60° at a scan rate of 10° min^–1^. The crystallinity index (CI) was calculated according to eq 2

2 $$\mathrm{CI}\;\left(\%\right)=\frac{A_{c}}{A_{c}+A_{a}}\times 100$$

where *A*_c_ and *A*_a_ are the integrated areas assigned to the crystalline and amorphous contributions, respectively, obtained by peak deconvolution using OriginLab software (OriginLab Corporation, Northampton, MA, USA).

#### 2.4.7 Mechanical Testing

Tensile tests were performed according to ASTM D882–18 using a computer-controlled universal testing machine (IP-90COM, IMPAC, São Paulo, Brazil). To mitigate premature grip failures, specimens were prepared as rectangular strips with a smooth, slightly narrowed gauge region. The overall specimen length was *L* = 100 mm and a nominal width of *w* = 20 mm. The initial grip separation was *L*_0_ = 50 mm, and the crosshead speed was 2 mm min^–1^. Film thickness was measured in the gauge region using a digital caliper (Mitutoyo, Kawasaki, Japan) with a resolution of 0.001 mm. The load cell provided a force resolution of 0.1 N and an accuracy of ±0.5%. Three specimens were tested per formulation, and results are reported as mean ± standard deviation. All tests were carried out at 23 °C (±2 °C) and 60% (±5%) relative humidity.

### 2.5 Statistical Analysis of Tensile Tests

Statistical analyses were performed separately for each mechanical descriptor (*F*_max_, distance at rupture, maximum elongation, ultimate tensile strength, and Young’s modulus). To test for an overall formulation effect, a one-way analysis of variance (ANOVA) was first applied using α = 0.05.

When a significant effect was detected, pairwise post hoc comparisons between formulations were carried out using Welch’s two-sample *t*-tests (two-sided), which do not assume equal variances. To control the family wise error rate across multiple comparisons, *p*-values were adjusted using the Holm step-down procedure. Differences were considered statistically significant when the adjusted *p*-value was below 0.05.

Statistically homogeneous groups within each column were identified based on the Holm-adjusted pairwise tests and denoted by superscript letters. Values that share at least one letter are not significantly different, whereas values with no letter in common differ significantly at α = 0.05.

## 3 Results and Discussion

### 3.1 Macroscopic Appearance

Figure 1 shows the macroscopic appearance of the as-prepared cellulose-based hydrogel films. The neat cellulose film (CEL) appears as the palest sample, exhibiting slight surface heterogeneities rather than a fully uniform surface, and serves as the visual baseline for assessing the effect of the additives.

**Figure 1:**
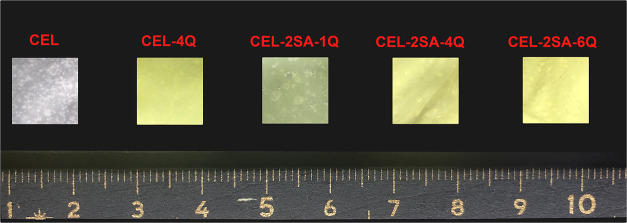
Representative photographs of the hydrogel films prepared in this study, illustrating the macroscopic appearance of real specimens with varying sodium alginate and quercetin contents. From left to right: CEL (100% cellulose), CEL-4Q (96% cellulose, 4% quercetin), CEL-2SA-1Q (97% cellulose, 2% sodium alginate, 1% quercetin), CEL-2SA-4Q (94% cellulose, 2% sodium alginate, 4% quercetin), and CEL-2SA-6Q (92% cellulose, 2% sodium alginate, 6% quercetin). The ruler at the bottom provides a scale reference in cm.

Incorporating quercetin without alginate (CEL-4Q) produces an evident yellowish coloration, consistent with the intrinsic chromophore of quercetin, and a macroscopically continuous surface appearance.

When sodium alginate is introduced at a fixed level (2%), the films (CEL-2SA-1Q, CEL-2SA-4Q, and CEL-2SA-6Q) display a progressively stronger yellow tone as quercetin loading increases from 1% to 6%. At the same time, the overall visual uniformity appears to change, with more noticeable macroscopic heterogeneities in the intermediate formulation (CEL-2SA-1Q) and a more continuous visual appearance in the higher-quercetin alginate films (CEL-2SA-4Q and CEL-2SA-6Q).

Overall, these photographs indicate that quercetin primarily governs the color variation, while the presence of alginate influences the macroscopic appearance of the films. It should be noted that such visual observations are qualitative and do not constitute direct evidence of miscibility or molecular-level interactions. A more detailed assessment of surface heterogeneity is presented in the following section based on FE-SEM analysis.

### 3.2 Surface Morphology by FE-SEM

Figure 2 presents FE-SEM micrographs of the film surfaces, allowing the macroscopic features observed in the photographs to be examined at the microscale.

**Figure 2:**
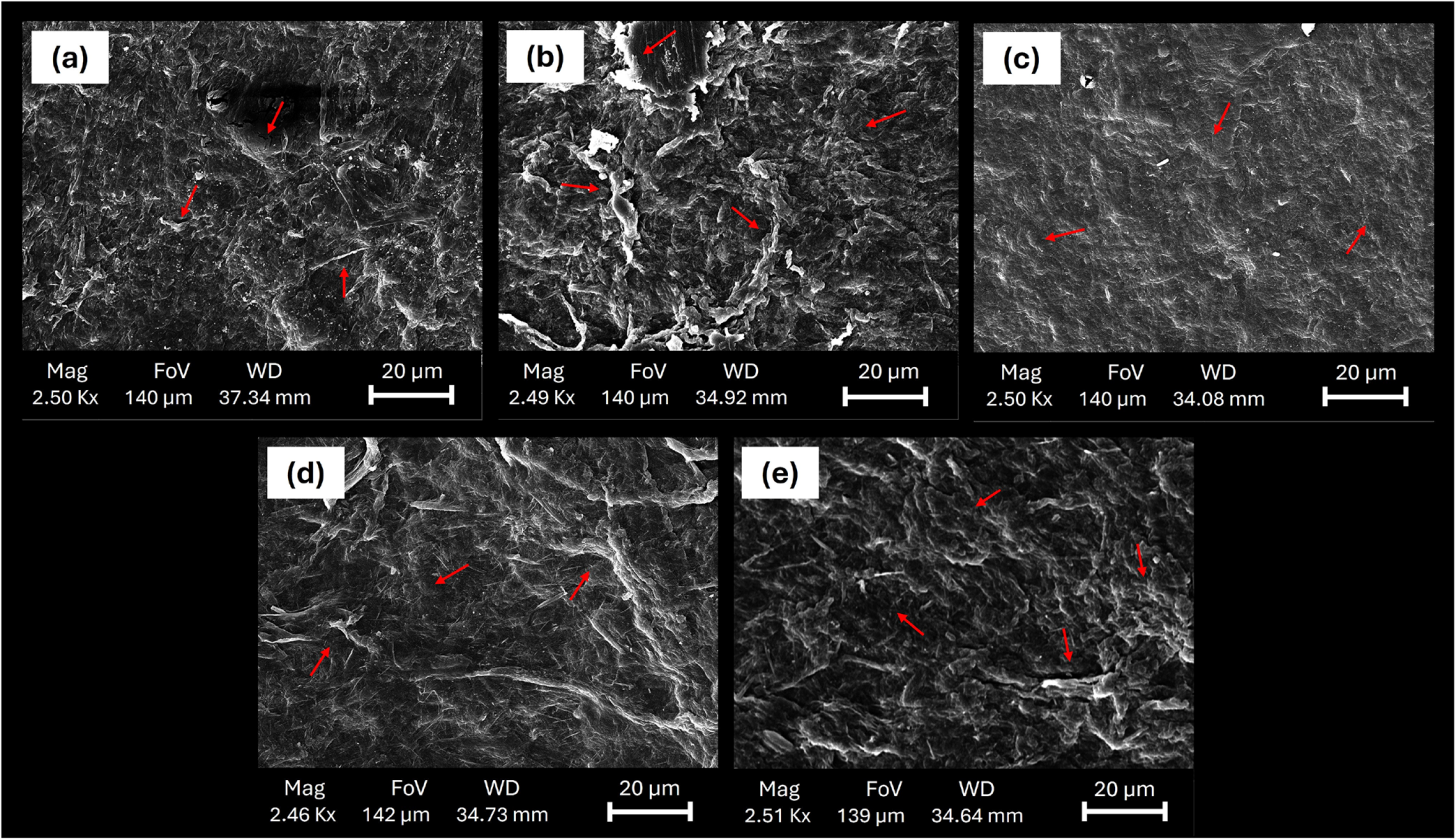
FE-SEM surface micrographs of the cellulose-based hydrogel films with varying sodium alginate and quercetin contents: (a) CEL, (b) CEL-4Q, (c) CEL-2SA-1Q, (d) CEL-2SA-4Q, and (e) CEL-2SA-6Q. Arrows indicate representative surface domains and textural heterogeneities. Scale bar: 20 μm.

In Figure 2a, the CEL surface shows a heterogeneous fibrillar and lamellar texture, which is commonly reported for regenerated cellulose systems and reflects the consolidation of a hydrogen-bonded polysaccharide network during drying.^24,25^

In CEL-4Q (Figure 2b), the surface becomes visibly rougher and more heterogeneous, with more frequent irregular domains. This behavior is consistent with reports on quercetin-containing polymer films, where limited miscibility and local aggregation of the rigid polyphenolic phase can generate surface-associated quercetin-rich domains and microstructural constraints that manifest as increased surface irregularity in SEM.^26^

However, when sodium alginate is incorporated at 2% (Figure 2c), the surface of the CEL-2SA-1Q film becomes more compact and visually more uniform than that of CEL-4Q, while preserving the characteristic fibrillar background of the cellulose matrix. This morphology is consistent with stronger polysaccharide-polysaccharide association and with a highly interconnected polysaccharide organization, in which alginate chains may occupy interfibrillar regions and reinforce cohesive packing through hydrogen bonding and carboxylate-mediated interactions.^27^

Evidence supporting this interpretation is provided by the FTIR spectra discussed in the next section, particularly the changes in the O–H stretching envelope and in the carboxylate and aromatic regions. Such an organization may reduce interfacial voids and hinder the development of surface-scale discontinuities. This interpretation can also be supported by corresponding trends in bulk responses, such as increased stiffness and strength, enhanced hydration,^28^ and lower optical scattering,^29^ as discussed in the next sections.

With a higher quercetin loading at the same sodium alginate content (CEL-2SA-4Q), the surface develops more pronounced, elongated quercetin-rich domains, visible as linear features highlighted by the red arrows in Figure 2d. This trend intensifies in CEL-2SA-6Q (Figure 2e), suggesting an increased tendency for quercetin aggregation or surface-enriched regions at higher loadings. Nevertheless, even at the highest quercetin content, the surface remains continuous (Figure 2e), without evidence of extensive cracking or macroscopic phase separation.

The observed morphological features can be directly related to the dispersion state of quercetin within the polymeric matrix. At low quercetin content (CEL-2SA-1Q), the relatively smooth and compact surface suggests a more homogeneous dispersion, likely facilitated by intermolecular interactions between quercetin and the cellulose-alginate network, which can partially suppress its intrinsic crystallinity. In contrast, as the quercetin content increases (CEL-2SA-4Q and CEL-2SA-6Q), the emergence of elongated and irregular domains indicates the formation of quercetin-rich regions, suggesting partial aggregation and phase heterogeneity due to the limited dispersion capacity of the matrix.

### 3.3 Fourier Transform Infrared Spectroscopy (FTIR)

Following the surface analysis, Figure 3 presents the FTIR spectra of the cellulose-based composite hydrogel films, aiming to determine whether the formulation-dependent surface textures are accompanied by changes in chemical signatures and intermolecular interactions.

**Figure 3:**
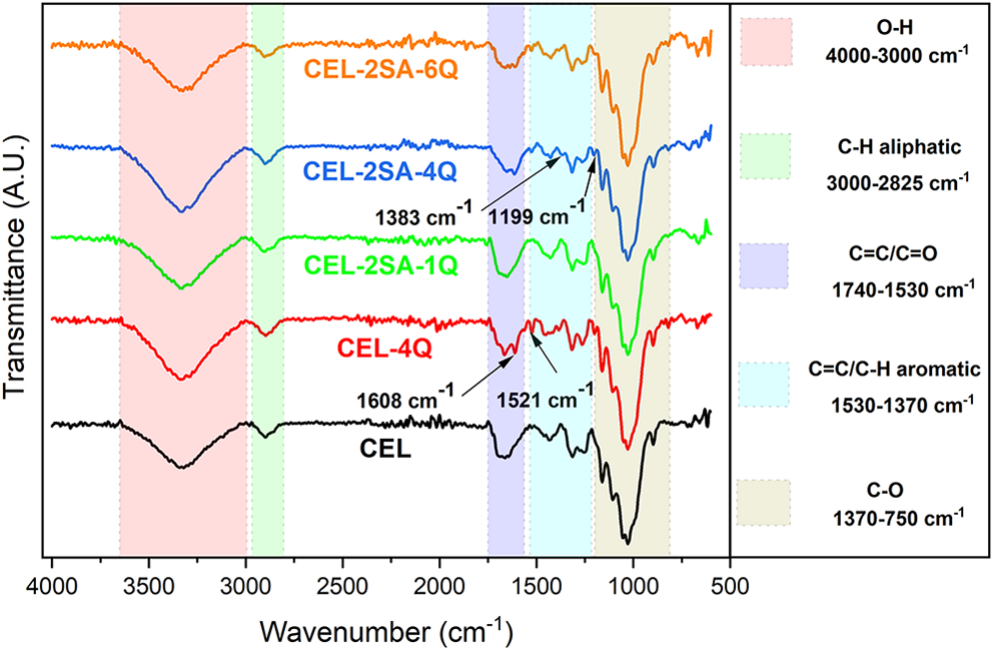
FTIR spectra of the cellulose-based hydrogel films with varying sodium alginate and quercetin contents. Shaded regions highlight the main vibrational bands assigned to O–H stretching (4000–3000 cm^–1^), aliphatic C–H stretching (3000–2825 cm^–1^), C=C and C=O vibrations (1740–1530 cm^–1^), aromatic C=C/C–H vibrations (1530–1370 cm^–1^), and C–O vibrations (1370–750 cm^–1^). Arrows indicate representative bands associated with quercetin incorporation.

In all formulations, a broad band in the 4000–3000 cm^–1^ range is observed and assigned to O–H stretching, reflecting extensive hydrogen bonding within the polysaccharide matrix (cellulose and sodium alginate)^30,31^ as well as contributions from the phenolic groups of quercetin.^32^ Aliphatic C–H stretching modes appear between 3000 and 2825 cm^–1^, as expected for polysaccharide backbones.^33^

The fingerprint region is governed by overlapping vibrations from the polysaccharide matrix and quercetin. Bands in the 1740–1530 cm^–1^ range include carbonyl stretching (mainly C=O, when present) and strong carboxylate stretching from alginate (COO^–^, typically with an asymmetric stretch near ∼1600 cm^–1^). The 1530–1370 cm^–1^ region is dominated by polysaccharide C–H bending modes, with additional aromatic-ring contributions from quercetin (mainly C=C ring stretching and in-plane C–H bending) when quercetin is present. Finally, the 1370–750 cm^–1^ region is primarily associated with backbone C–O and glycosidic C–O–C stretching vibrations of cellulose and alginate, with extra phenolic C– O contributions from quercetin.^34^

Against this common polysaccharide background, the clearest formulation-dependent changes are linked to the presence of quercetin. In CEL-4Q and in the alginate-containing films with higher quercetin contents (CEL-2SA-4Q and CEL-2SA-6Q), quercetin-related aromatic ring vibrations become progressively more evident, particularly near 1608 and 1521 cm^–1^, which are commonly assigned to aromatic C=C stretching modes. Additional features near 1383 cm^–1^ are consistent with C–H bending (single bond environments), while the band around 1199 cm^–1^ is attributed mainly to C–O stretching of phenolic and alcohol groups (single C–O bonds).^34^ The absence of clear new bands suggesting the formation of new covalent linkages supports the conclusion that quercetin is incorporated predominantly through noncovalent interactions, mainly hydrogen bonding and secondary associations within the polysaccharide network.

The exception is CEL-2SA-1Q, in which the bands at 1608, 1521, 1383, and 1199 cm^–1^ are not clearly resolved as discrete features. Instead, CEL-2SA-1Q displays a slightly broader O–H stretching envelope and a more pronounced, broad contribution in the 1650–1550 cm^–1^ region, consistent with greater adsorption of ambient moisture and a higher fraction of hydrogen-bonded (bound) water in the alginate-containing polysaccharide network.

The influence of sodium alginate is mainly reflected in subtle changes in band definition and relative intensities, rather than in the emergence of new spectral features, particularly in the 1740–1530 cm^–1^ and 1200–900 cm^–1^ regions where alginate and cellulose vibrations overlap. This behavior is consistent with the presence of strong intermolecular interactions between cellulose and alginate, which may lead to a highly interconnected polysaccharide network. In this context, the system can be interpreted as exhibiting features compatible with a highly interconnected polysaccharide organization, in which alginate chains may be distributed within the cellulose fibrillar scaffold and contribute to an increased density of physical junctions.^35^

Such an organization provides a chemically consistent explanation for the more compact and uniform surface observed by SEM for CEL-2SA-1Q relative to CEL-4Q, and it is also consistent with the improved mechanical integrity observed for the alginate-containing formulation.^28^ Moreover, the broader O–H envelope observed for CEL-2SA-1Q is compatible with a higher fraction of hydrogen-bonded water associated with the hydrated polysaccharide network, as discussed in the next sections.

### 3.4 X-ray Diffraction (XRD)

Figure 4 presents the XRD patterns (Figure 4a) and the corresponding crystallinity indices (Figure 4b) of the analyzed samples. The diffractograms show the characteristic reflections of native cellulose I-β, the predominant polymorph in higher plants, with a prominent reflection at 2θ ≈22.7° assigned to the (200) plane for measurements performed with Cu Kα radiation.^36^ Across the series, the relative prominence of the cellulose (200) reflection with respect to the amorphous background decreases, which is consistent with the crystallinity index trend (66.9% for CEL to 54.6% for CEL-2SA-6Q; Figure 4b). The individual deconvolution fittings of the XRD profiles, used to estimate the crystallinity index of each formulation, are provided in the Supporting Information.

**Figure 4:**
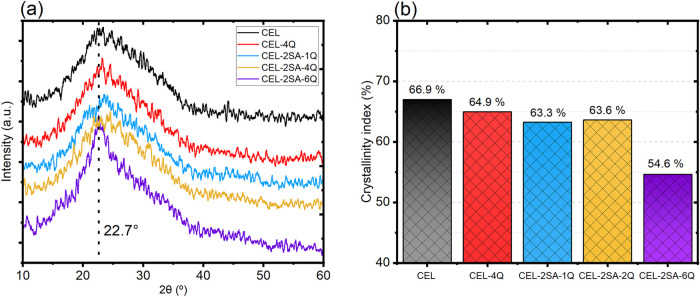
(a) X-ray diffraction (XRD) patterns of cellulose-based hydrogel films with different formulations, showing the typical Cellulose I profile (dashed lines) and formulation-dependent changes in diffraction intensity and halo shape across 2θ = 10–60°. (b) Crystallinity index determined from the XRD patterns for each formulation.

This behavior is expected when sodium alginate is blended with cellulose. Under typical processing conditions, alginate does not cocrystallize with cellulose because differences in chain conformation and functional group spacing hinder accommodation within the same crystal lattice.^28^ Instead, alginate is preferentially located in the interfibrillar or interdomain regions, increasing the amorphous contribution and effectively diluting the bulk crystallinity signature of the cellulose rich phase.^18^ Superimposed on this matrix effect, quercetin can further perturb cellulose packing by establishing competitive intermolecular interactions with hydroxyl and carboxylate groups, which is reflected by the lower crystallinity index at higher loading (notably CEL-2SA-6Q).^37^

Regarding the crystallinity results, CEL shows a crystallinity index of 66.9%, placing it toward the upper end of values commonly reported for semicrystalline cellulose-based materials. As a reference point, microcrystalline cellulose typically exhibits crystallinity indices in the 65–80% range, whereas regenerated cellulose films often display lower values (approximately 40–60%), depending on the regeneration route and processing history.^38^ The relatively high crystallinity of CEL suggests that the mechanical processing used in this work promoted fiber dispersion while largely preserving the crystalline domains of the cellulose microfibrils.^39^

Comparing CEL with CEL-4Q, we observe a slight decrease in crystallinity from 66.9% to 64.9%. This trend is consistent with reports in polymer and polysaccharide-based films where the incorporation of low-molecular-weight bioactive compounds leads to an interfacial disordering effect, reducing the apparent crystalline contribution by perturbing the host hydrogen-bond network.^40^ In our system, quercetin can act as a competing hydrogen-bonding agent, since its phenolic hydroxyl groups may partially disrupt cellulose-cellulose associations, thereby hindering optimal chain packing and slightly lowering the crystalline order.^41^

Despite a 4-fold increase in quercetin content from CEL-2SA-1Q to CEL-2SA-4Q, the crystallinity index remains essentially unchanged (approximately 63%). However, at a fixed quercetin content (4 wt %), adding 2 wt % sodium alginate leads to a further, albeit modest, decrease in crystallinity (CEL-4Q: 64.9% vs CEL-2SA-4Q: 63.6%). This trend is consistent with cellulose-alginate blends in which alginate does not participate in the cellulose I lattice; instead, it preferentially occupies noncrystalline regions, increasing the amorphous contribution and limiting the development of cellulose ordering during regeneration and drying.^42^

In addition, strong intermolecular interactions between alginate carboxylate groups and cellulose hydroxyls have been reported in alginate-nanocellulose composite films,^43^ supporting the interpretation that alginate can interact with cellulose surfaces in the amorphous domains. Finally, cellulose-alginate networks have also been used as matrices for quercetin incorporation,^44^ indicating that such polysaccharide interactions can persist in the presence of phenolic additives.

The most striking difference across the series is the pronounced crystallinity drop observed for CEL-2SA-6Q (CI = 54.6%). Up to 4% quercetin (CEL-2SA-4Q), the crystallinity remains comparatively stable, suggesting that the additive is mainly accommodated within amorphous and interfacial regions of the cellulose rich network. At 6% loading, however, the additional quercetin likely exceeds the capacity for molecular level dispersion, favoring the formation of phenolic rich domains that interfere with interchain packing and reduce the coherent crystalline fraction. Similar concentration dependent losses of crystallinity and diffraction peak broadening have been reported in polysaccharide based films enriched with phenolic extracts, and are commonly attributed to polyphenol polymer hydrogen bonding that disrupts ordered chain arrangement and limits crystal growth.^45,46^

### 3.5 UV–Vis Spectroscopy

To complement the structural and physicochemical characterization discussed above, Figure 5 summarizes how the different formulations modulate light attenuation across the UV and visible ranges. This analysis helps distinguish absorption associated with chromophores from light losses caused by scattering, and thus interpret both color development and optical screening performance of the cellulose-quercetin-alginate hydrogel films.

**Figure 5:**
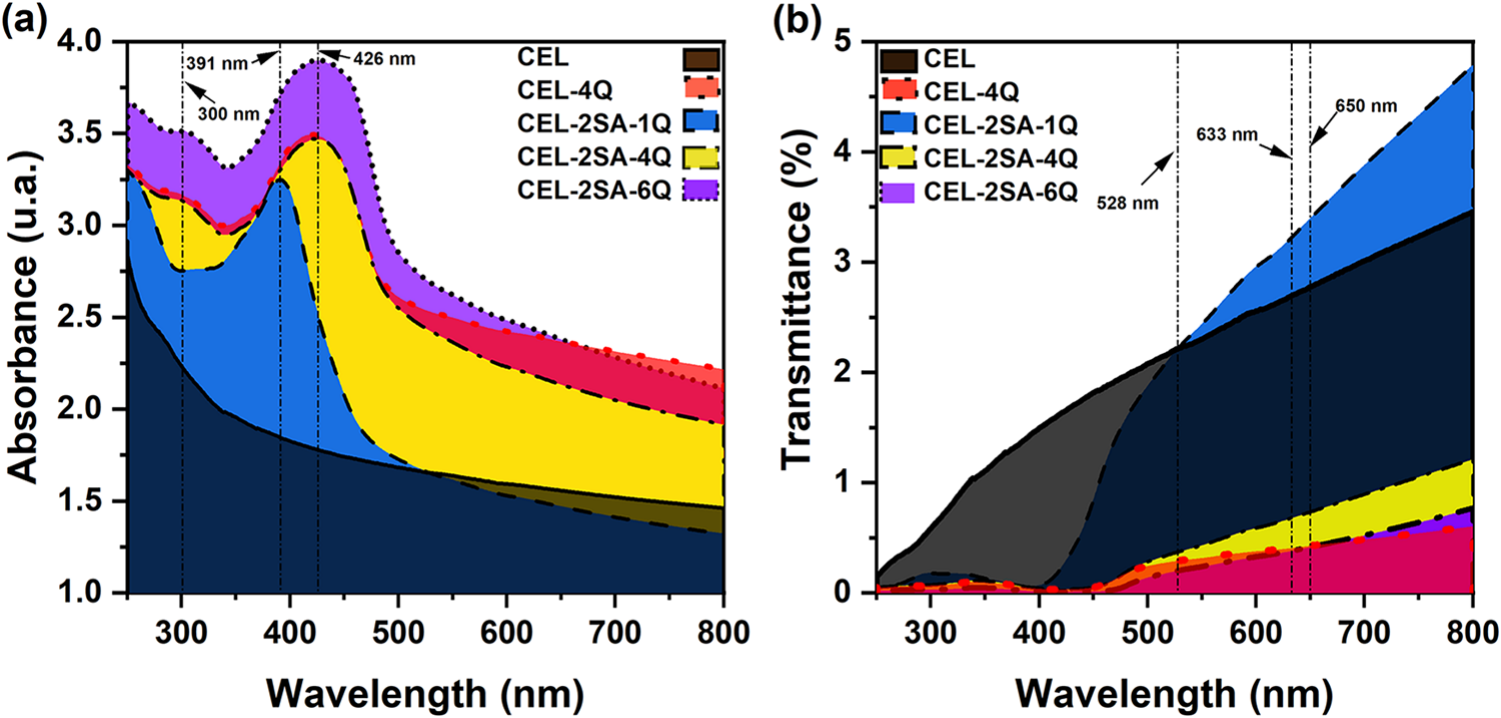
UV–vis spectra of the cellulose-based hydrogel films (CEL, CEL-4Q, CEL-2SA-1Q, CEL-2SA-4Q, and CEL-2SA-6Q): (a) absorbance and (b) transmittance in the 250–800 nm range. The vertical dashed lines indicate representative wavelengths highlighted for comparison (300, 391, and 426 nm in panel a; 528, 633, and 650 nm in panel b).

The control film CEL exhibits the lowest absorbance (Figure 5a) across the entire spectral region, which is consistent with the absence of conjugated chromophores. This behavior agrees with the FTIR results, which showed a predominance of absorption bands attributed to O–H and C–H/C-O vibrations of cellulose, as well as with the XRD data indicating a high degree of crystallinity and the SEM images revealing a continuous fibrillar structure. At 633 nm, Figure 5b, CEL shows intermediate transmittance (∼2.69%), which is typical of a cellulosic matrix without aromatic light absorbers.^47^

The introduction of quercetin in CEL-4Q leads to the appearance of a new absorption band extending toward the visible region, with a maximum at approximately 426 nm, attributed to π → π* electronic transitions of the conjugated aromatic system of quercetin. The increase in absorbance corroborates the FTIR data, reflecting a greater contribution of phenolic groups, mainly associated with vibrational modes of the aromatic ring (C=C, C–H, and C–O), to intermolecular interactions. Vilera et al.^48^ reported that phenolic compounds can absorb UV light due to their benzene rings; thus, the enhanced contribution of these groups observed in FTIR supports the higher content of benzene rings and the consequent increase in UV absorption. Moreover, the higher surface roughness observed by SEM also contributes to the increased absorbance values.^49^ At 633 nm, the low transmittance of CEL-4Q (0.40%) confirms the strong influence of quercetin on reducing visible-light transmittance.

For CEL-2SA-1Q, which contains sodium alginate in the matrix and a low concentration of quercetin, the absorption peak shifts to approximately 391 nm, suggesting a change in the electronic environment of the quercetin chromophoric groups and indicating the formation of a more interactive polymeric network between alginate and cellulose. XRD data, which indicate a slight decrease in crystallinity, support the idea that molecular-level structural reorganization affects the optical interactions of the film.^50,51^ At 633 nm, CEL-2SA-1Q exhibits the highest transmittance among the samples (3.22%), demonstrating that the combination of alginate with a low quercetin content results in a more optically transparent matrix in this region.

Increasing quercetin levels in CEL-2SA-4Q and CEL-2SA-6Q result in a gradual increase in absorbance intensity across the entire UV–vis range, indicating a higher presence of conjugated aromatic systems. The spectral response of these samples becomes increasingly dominated by the electronic transitions of quercetin, with absorption maxima tending toward 426 nm. This behavior is also consistent with FTIR data, which indicate more pronounced contributions from aromatic and oxygenated functional groups, as well as with XRD results revealing further modulation of the balance between crystalline and amorphous phases in the cellulose-based matrix. Transmittance spectra provide additional insight into the combined effects of composition and structure; at 633 nm, the low transmittance values observed for CEL-2SA-4Q (0.68%) and CEL-2SA-6Q (0.38%) confirm the increasing effect of quercetin on visible-light attenuation.

The transition observed at 528 nm (Figure 5b) between CEL and CEL-2SA-1Q corresponds to a spectral region where the dominant optical response shifts from chromophore-driven absorption to matrix-dependent behavior. At shorter wavelengths, the optical response is primarily governed by π → π* electronic transitions of quercetin, as evidenced by the characteristic absorption band near 426 nm and its dependence on quercetin content. As the wavelength increases, the contribution of these electronic transitions diminishes, and differences in matrix organization and intermolecular interactions within the cellulose-alginate network become more significant, consistent with the structural features observed by FTIR, XRD, and SEM. Similarly, the transition at 650 nm between CEL-4Q and CEL-2SA-6Q reflects the balance between quercetin concentration and the polymeric network.

Overall, the UV–vis results indicate that the optical properties of the films depend not only on the amount of quercetin incorporated, but also on the organization of the cellulose-alginate matrix and the intermolecular interactions occurring within the system. These findings are consistent with the structural data obtained from FTIR, XRD, and FE-SEM, highlighting the influence of matrix composition and molecular organization on the modulation of the films’ optical response.

### 3.6 Wetting Dynamics and Water Uptake

Surface wetting and bulk water sorption of the cellulose based films were assessed by time dependent contact angle measurements (Figure 6a) and gravimetric water uptake (Figure 6b), respectively. Because these experiments probe distinct length scales, their joint interpretation helps separate near surface spreading and penetration from bulk swelling controlled by network chemistry.

**Figure 6:**
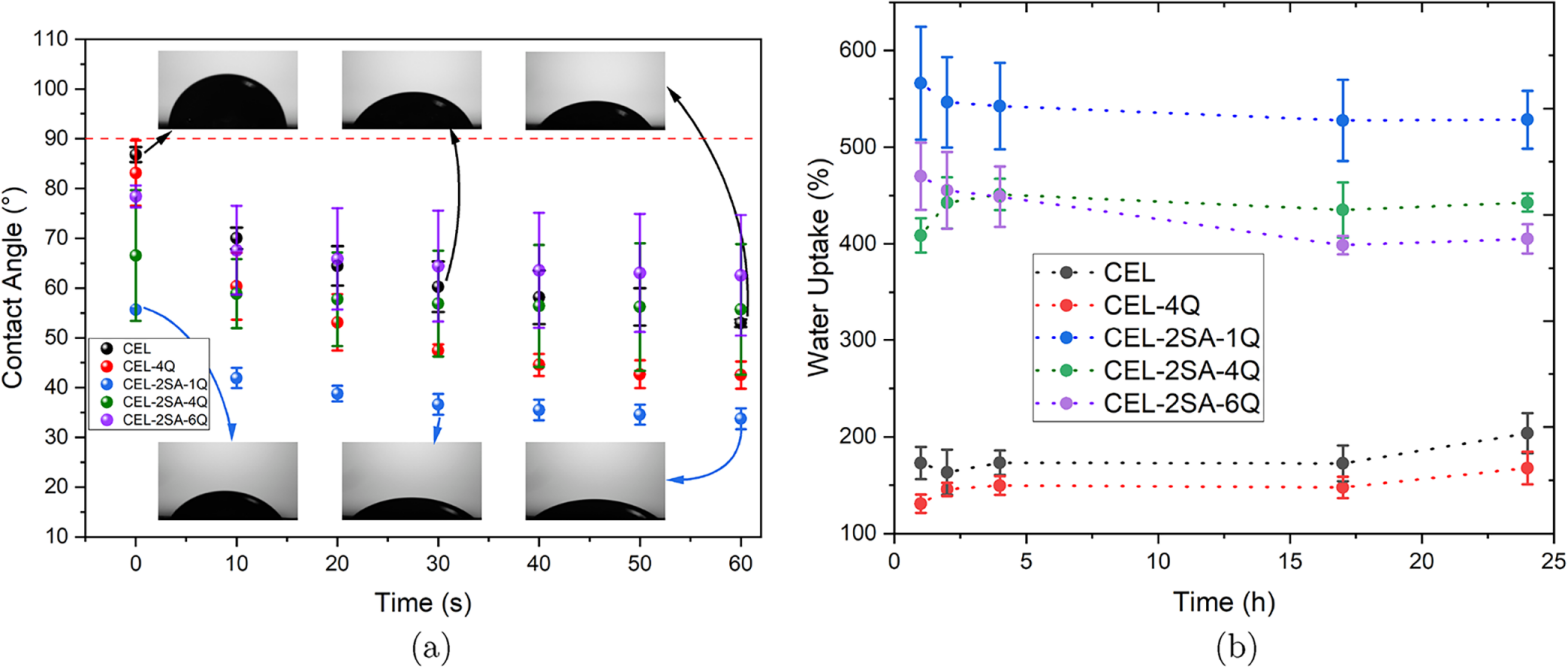
Wetting and water sorption behavior of cellulose-based films: (a) time-dependent water contact angle and representative droplet images, and (b) gravimetric water uptake as a function of immersion time for CEL, CEL-4Q, CEL-2SA-1Q, CEL-2SA-4Q, and CEL-2SA-6Q.

Neat cellulose (CEL) exhibits a high initial contact angle close to 90° and a gradual decrease within 60 s (Figure 6a), consistent with limited short time wetting. In agreement, CEL shows the lowest water uptake in the series, rising from approximately 160–180% at early times to about 200% after 24 h (Figure 6b). These results are consistent with a densely hydrogen bonded cellulosic matrix and the heterogeneous fibrillar surface observed by SEM.

Adding quercetin without alginate (CEL-4Q) slightly accelerates the contact angle decay relative to CEL and increases the water uptake to roughly 130–170% (Figure 6a,b). The SEM evidence of increased roughness and particle like domains supports the presence of heterogeneous regions that can promote local wetting pathways while still limiting bulk swelling when the matrix remains predominantly cellulosic.

A marked change is observed when sodium alginate is introduced. CEL-2SA-1Q displays rapid wetting, reaching contact angles in the 35–40° range by 60 s (Figure 6a), and exhibits the highest water uptake, approximately 520–570% across the test window (Figure 6b). Although SEM indicates a more compact and homogeneous surface, the strong hydrophilicity of alginate-containing networks likely dominates the water interaction through abundant polar functionalities, leading to both fast wetting and extensive bulk swelling.

Increasing quercetin content in alginate-containing films (CEL-2SA-4Q and CEL-2SA-6Q) slightly reduces the wetting rate and increases the contact angle relative to CEL-2SA-1Q (Figure 6a), while maintaining high water uptake levels, with CEL-2SA-4Q remaining around 430–450% and CEL-2SA-6Q around 400–450% over the immersion period (Figure 6b). The progressive increase in roughness and visible agglomeration in SEM at higher quercetin contents suggests that quercetin rich domains partially reduce the effective hydrophilic fraction and hinder water transport, moderating swelling without suppressing the dominant alginate driven sorption.

Thus, the results indicate that alginate is the primary driver of water affinity and bulk swelling in these systems, whereas quercetin acts as a secondary modulator that tunes wetting kinetics and sorption magnitude in a concentration dependent manner, consistent with the morphological trends observed by SEM.

These trends imply that, under wet conditions, the alginate-rich formulations may experience reduced dimensional stability and moisture-induced loss of mechanical integrity. Thus, the improved absorbency is accompanied by a practical trade-off between fluid uptake and structural stability during prolonged water exposure.

### 3.7 Uniaxial Tensile Strength Test

One of the most notable improvements observed upon incorporating sodium alginate and quercetin into cellulose films is the increase in mechanical strength. However, optimal performance depends on a careful balance between sodium alginate and quercetin contents, since their combined effects can alter stiffness and ductility as well as strength. Table 2 summarizes the tensile properties of the films, reported as mean ± standard deviation (SD). In addition, representative stress–strain curves (Figure 7) illustrate the differences in elastic response, strain hardening, and failure behavior among the formulations.

**Figure 7:**
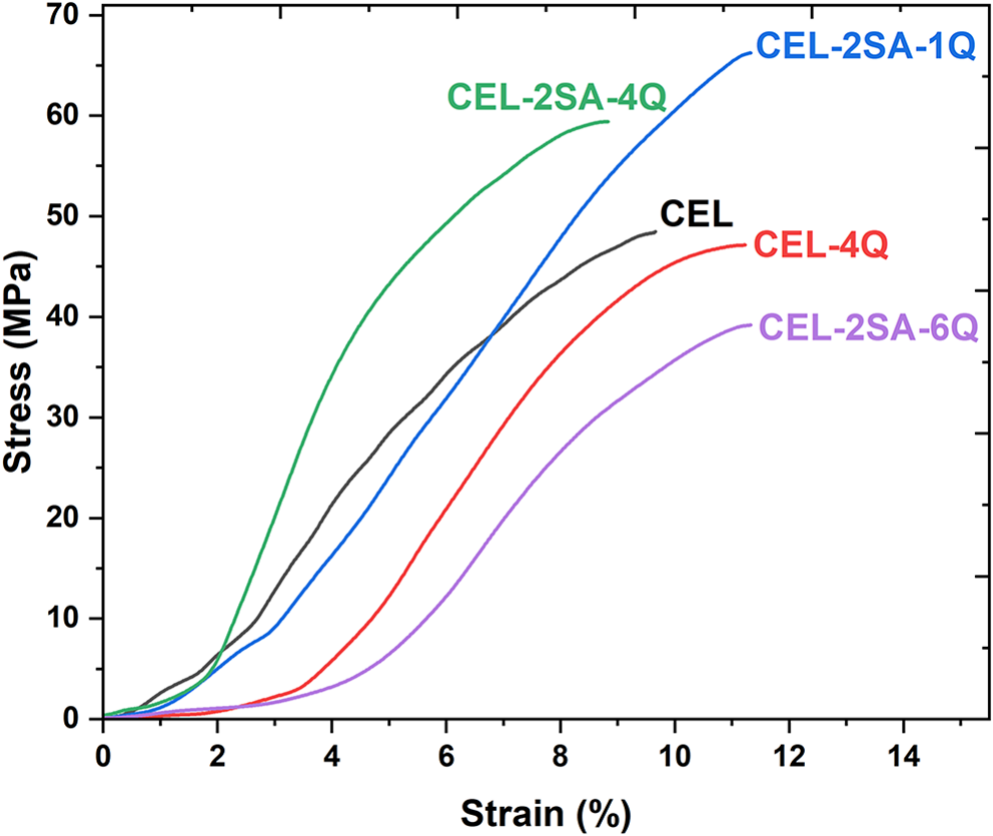
Representative stress–strain curves of the investigated formulations. Each curve corresponds to a representative specimen, as replicate tests exhibited similar mechanical behavior. Statistical tensile parameters are summarized in Table 2.

**Table 2:** Tensile Results (Mean ± SD)^a^

Sample	*F*_max_ (N)	Dist. rupture (mm)	Elong. max (%)	UTS (MPa)	Young’s modulus (MPa)
CEL	95.85 ± 14.81^*c*^	2.92 ± 0.22^*b*^	9.75 ± 0.32^*b*^	43.57 ± 6.73^*c*^	513.2 ± 29.7^*e*^
CEL-4Q	100.62 ± 15.06^*bc*^	3.37 ± 0.36^*ab*^	11.25 ± 2.48^*abc*^	45.74 ± 6.84^*bc*^	1267.8 ± 41.8^*b*^
CEL-2SA-1Q	147.85 ± 9.82^*a*^	3.40 ± 0.56^*ab*^	11.34 ± 2.18^*abc*^	67.20 ± 4.46^*a*^	1411.3 ± 18.3^*a*^
CEL-2SA-4Q	126.10 ± 10.11^*b*^	2.65 ± 0.07^*b*^	8.83 ± 0.24^*c*^	57.32 ± 4.60^*b*^	1041.3 ± 39.0^*c*^
CEL-2SA-6Q	86.20 ± 10.43^*c*^	3.57 ± 0.15^*a*^	11.89 ± 0.51^*a*^	39.18 ± 4.74^*c*^	631.5 ± 33.6^*d*^

a *F*_max_: maximum force; Dist. rupture: displacement at break; Elong. max: elongation at break (%); UTS: ultimate tensile strength and; Young’s modulus. Different superscript letters within the same column indicate statistically significant differences (α = 0.05).

Overall, the formulation significantly affects all mechanical metrics (as indicated by the different superscript letters within each column), revealing clear composition-structure–property relationships that are consistent with the microstructural trends observed by SEM (Figure 2) and the interaction signatures detected by FTIR (Figure 3), as well as with the crystallinity modulation measured by XRD (Figure 4).

The control film (CEL) exhibits the lowest stiffness (Young’s modulus) while maintaining moderate strength (UTS) and limited extensibility. This behavior is consistent with its relatively high crystallinity index (Figure 4) and the continuous fibrillar background seen in SEM (Figure 2a), reflecting a regenerated cellulose network consolidated mainly by cellulose-cellulose hydrogen bonding. This behavior is also reflected in the relatively moderate slope and gradual stress increase observed in the stress–strain curve (Figure 7).

Upon quercetin incorporation without alginate (CEL-4Q), the Young’s modulus increases markedly relative to CEL, whereas the changes in *F*_max_ and UTS are comparatively modest. This divergence suggests that quercetin primarily stiffens the network by introducing additional physical junctions and constraining chain mobility through noncovalent interactions, rather than acting as an effective reinforcement mechanism for ultimate strength. This interpretation is supported by FTIR, which indicates that quercetin is incorporated mainly via hydrogen bonding and secondary associations (Figure 3), and by SEM observations for the quercetin-containing surface, which becomes rougher and more heterogeneous, indicating local quercetin-rich domains that can limit strength gains by acting as stress concentrators (Figure 2b). Consistently, the stress–strain curve shows a steeper initial slope compared to CEL, confirming the increase in stiffness.

Introducing sodium alginate together with quercetin produces the strongest and stiffest formulation at intermediate loading (CEL-2SA-1Q), which shows the highest *F*_max_, UTS, and Young’s modulus among all samples. This improvement is consistent with a denser polysaccharide network in which alginate chains occupy noncrystalline or interfibrillar regions and may contribute to an increased density of physical junctions through hydrogen bonding and carboxylate-mediated interactions, as suggested by the FTIR changes in the O–H and carboxylate regions (Figure 3) and by the more compact morphology observed for alginate-containing films (Figure 2c).^27,35^

Notably, the crystallinity of the alginate-containing films remains near ∼63% up to 4% quercetin (Figure 4), indicating that the strength and stiffness gains observed for CEL-2SA-1Q are driven mainly by network densification and improved cohesion, rather than by an increase in crystalline fraction.

At higher quercetin loading with constant alginate content, however, the mechanical response shows a clear trade-off. CEL-2SA-4Q remains mechanically robust but displays lower *F*_max_, UTS, and modulus than CEL-2SA-1Q, together with the lowest elongation at break. This pattern is consistent with the emergence of more pronounced quercetin-rich elongated domains in SEM (Figure 2d), which likely increase microstructural constraint and reduce the ability of the network to dissipate strain, promoting earlier failure under tension.

Finally, CEL-2SA-6Q exhibits the most distinct shift in mechanical behavior: strength and stiffness decrease relative to the 1Q and 4Q formulations, while the distance at rupture and elongation increase. This transition is coherent with the pronounced drop in crystallinity at the highest quercetin loading (Figure 4), together with the stronger tendency for quercetin-rich domains in SEM (Figure 2e). In this regime, the additional quercetin likely exceeds the capacity for molecular-level dispersion and increasingly perturbs cellulose packing (as also suggested by the XRD trend), leading to a larger amorphous contribution and a softer, more deformable network. Similar concentration-dependent reductions in crystallinity and peak broadening have been reported for phenolic-enriched polysaccharide films and are commonly attributed to polyphenol–polymer hydrogen bonding that disrupts ordered packing and limits crystal growth.^45,46^

Taken together, the tensile data indicate that moderate quercetin incorporation in the presence of alginate (CEL-2SA-1Q) maximizes cohesive reinforcement (higher strength and stiffness), whereas excessive quercetin loading (CEL-2SA-6Q) shifts the balance toward reduced order (lower crystallinity and stiffness) but increased deformability, in agreement with the SEM and XRD evidence of stronger domain formation and structural disorder at the highest loading.

## 4 Conclusions

This study demonstrates that a ternary cellulose-sodium alginate-quercetin system can partially decouple the competing requirements of water affinity, UV–vis shielding, and mechanical robustness in fully biobased films. FTIR and XRD suggest predominantly noncovalent incorporation and are consistent with the presence of intermolecular interactions within a physically cross-linked polysaccharide network, while FE-SEM shows that moderate quercetin loadings preserve a continuous fibrillar structure, whereas higher contents promote phenolic-rich domains and reduced strain dissipation.

Functionally, quercetin governs UV–vis attenuation and color, while alginate drives hydration and wetting. The optimized composition (CEL-2SA-1Q, 97/2/1 wt %) achieved the best overall performance, combining high mechanical strength (UTS = 67.20 ± 4.46 MPa), enhanced hydrophilicity (contact angle ∼35° at 60 s), and a ∼3-fold increase in water uptake, while maintaining UV–vis transmittance below 5%.

These results suggest that synergistic polysaccharide interactions and controlled polyphenol incorporation can enable simultaneous mechanical reinforcement, hydration, and optical shielding, supporting the potential of the optimized formulation as an absorbent and photoprotective layer for sustainable packaging of high-moisture and light-sensitive products.

Future work will focus on translating this material into application-relevant formats and expanding the compositional design space to further optimize performance.

## Supplementary Material



## Data Availability

All data supporting the findings of this study are provided within the Article. Additional raw data (including original instrument outputs and analysis files) are available from the corresponding author upon reasonable request.

## Notes

Additional XRD peak fitting results, including full deconvolution profiles (Figure S1) (ZIP)

## Abbreviations

ANOVA: analysis of variance
ASTM: ASTM International
ATR-FTIR: attenuated total reflectance Fourier transform infrared spectroscopy
CI: crystallinity index
FE-SEM: field emission scanning electron microscopy
FTIR: Fourier transform infrared spectroscopy
IPN: interpenetrating polymer network
PE: polyethylene
PP: polypropylene
PS: polystyrene
PET: poly­(ethylene terephthalate)
PVC: poly­(vinyl chloride)
Q: quercetin
RH: relative humidity
SA: sodium alginate
SD: standard deviation
SDG: Sustainable Development Goal
SDGs: Sustainable Development Goals
UTS: ultimate tensile strength
UV: ultraviolet
UV–vis: ultraviolet visible spectroscopy
WU: water uptake
XRD: X-ray diffraction
*F*_max_: maximum force
