# Supplementary material for: Balancing Water Uptake, UV Visible Screening, and Mechanical Strength in Cellulose Alginate Quercetin Hydrogel Films
Source: ACS Omega. 2026 May 25;11(22):32790–801. doi: 10.1021/acsomega.6c01788 (PMC13261450; doi:10.1021/acsomega.6c01788)
Supplement: Supplementary file 1 [file ao6c01788_si_001.zip › sup-info.pdf]

Supporting Information

Balancing Water Uptake, UV Visible Screening, and Mechanical Strength in Cellulose Alginate Quercetin Hydrogel Films

Sarah Kalli Silva da Silva,[†] André Lamounier Caixeta,[†] Marlon Bender Bueno Rodrigues,[†] Bruna Bicca Fernandes,[†] Patricia Oliveira Schmitt,[†] Lincoln Audrew Cordeiro,[†] Ivandra Ignês de Santi,[†] Evandro Piva,[‡] Neftali Lenin Villarreal Carreño,[†] Amanda Dantas de Oliveira,[†] Cesar Aguzzoli,[¶] Everton Granemann Souza,[*,§] André Luiz Missio[*,†] and Chiara das Dores do Nascimento[†,‖]

[†]Graduate Program in Materials Science and Engineering (PPGCEM), Federal University of Pelotas (UFPel), Pelotas, RS, 96010-610, Brazil

[‡]Graduate Program in Dentistry (PPGO), School of Dentistry, Federal University of Pelotas (UFPel), Pelotas, RS, 96015-560, Brazil

[¶]Graduate Program in Materials Science and Engineering (PPGMAT), University of Caxias do Sul, Caxias do Sul, RS, 95070-560, Brazil

[§]Graduate Program in Electronics and Computer Engineering (MEEC), Catholic University of Pelotas (UCPel), Pelotas, RS, 96015-560, Brazil

[‖]Graduate Program in Sciences and Technologies in Education (PPGCITED), Federal Institute of Education, Science and Technology of Rio Grande do Sul (IFSul), Pelotas, RS, 96060-290, Brazil

*Corresponding author: Prof. Everton Granemann Souza: everton.granemann@ucpel.edu.br;
*Corresponding author: Prof. Andre Luiz Missio: andre.missio@ufpel.edu.br

Additional XRD Analysis

Figure S1 shows the detailed peak deconvolution used for crystallinity index (CI) calculation in all formulations.

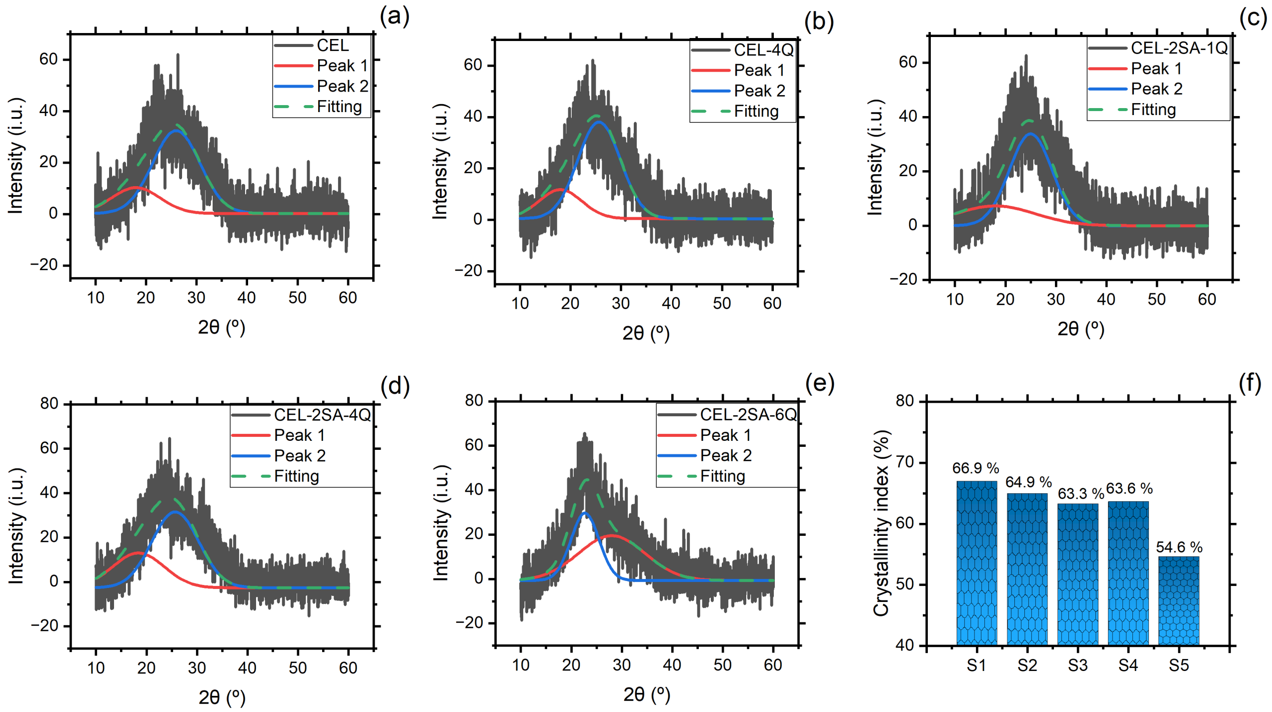

Figure 1: X-ray diffraction (XRD) analysis and crystallinity quantification of the cellulose-based hydrogel films. (a-e) Deconvolution of the diffraction patterns for samples CEL, CEL-4Q, CEL-2SA-1Q, CEL-2SA-4Q, and CEL-2SA-6Q. The profiles were fitted using Gaussian functions, where Peak 1 (red) corresponds to the amorphous halo (18°) and Peak 2 (blue) represents the crystalline contribution associated with the (200) plane of cellulose I (22-23°). The dashed green line indicates the overall fitting, showing good agreement with the experimental profiles across the entire 2θ range. (f) Resulting crystallinity index (CI, %) calculated from the area integration of the deconvoluted peaks.
