# Supplementary figures and images for: Balancing Water Uptake, UV Visible Screening, and Mechanical Strength in Cellulose Alginate Quercetin Hydrogel Films
Source: ACS Omega. 2026 May 25;11(22):32790–801. doi: 10.1021/acsomega.6c01788 (PMC13261450; doi:10.1021/acsomega.6c01788)

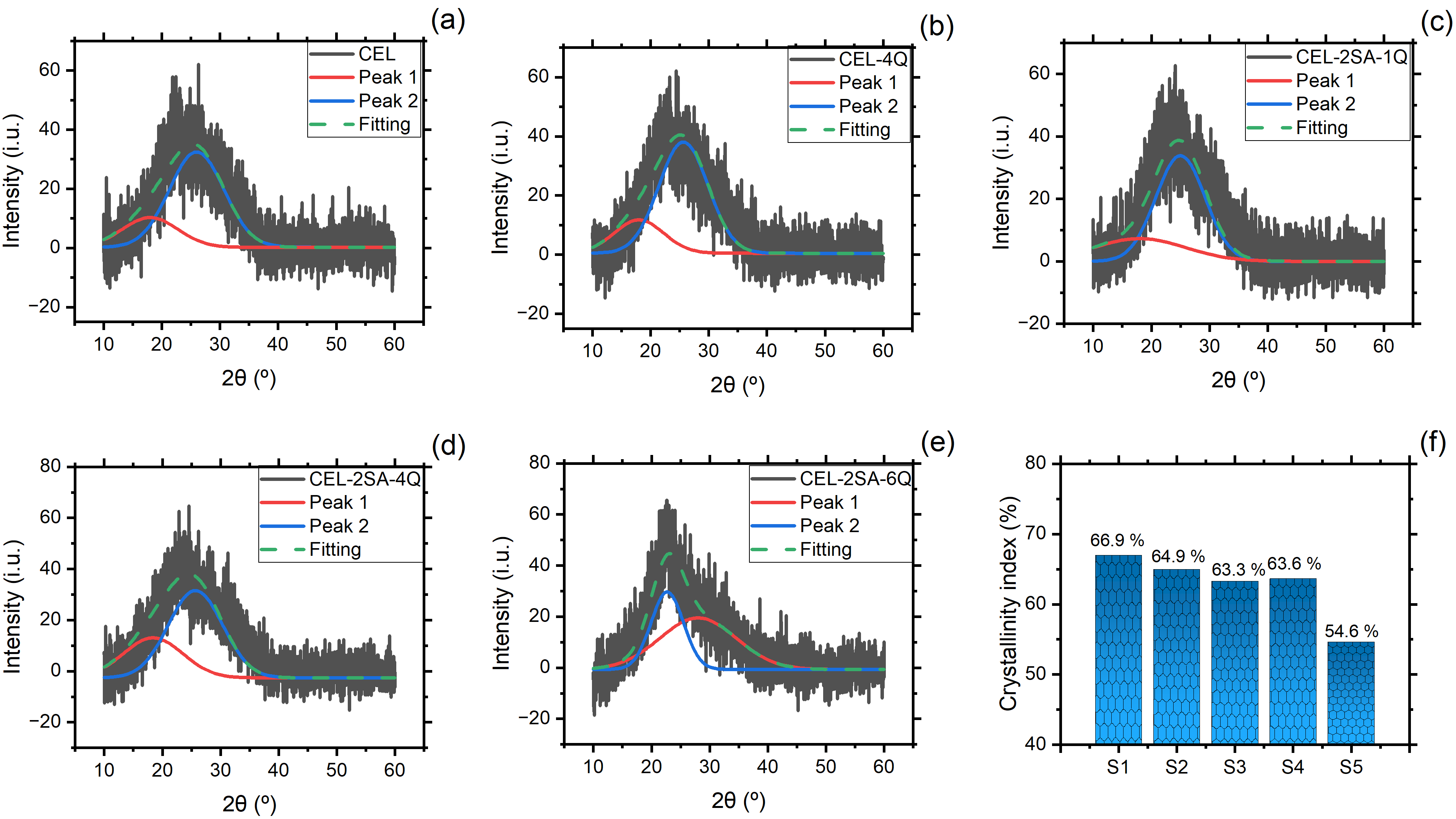

Supplement: Supplementary file 1 [file ao6c01788_si_001.zip › xrd-sup.png]
